# MicroRNA dysregulation influences growth hormone signaling

**DOI:** 10.18632/aging.102145

**Published:** 2019-08-06

**Authors:** Amy E. Elias, Jill A. Kreiling

**Affiliations:** 1 Department of Molecular Biology, Cell Biology and Biochemistry, and Center on the Biology of Aging, Brown University, Providence, RI 02903, USA

**Keywords:** aging, miRNAs, growth hormone receptor, growth hormone signaling, mouse, liver

The somatotropic axis has been implicated in regulating healthspan and lifespan in a wide range of organisms from invertebrates to humans [1]. The somatotropic axis consists of growth hormone releasing hormone, growth hormone (GH), and insulin-like growth factor 1 (IGF-1) that work together to regulate cell metabolism, survival and growth. In mammals, GH is released into the circulation by the pituitary gland, and its primary site of action is the liver, where it activates the growth hormone receptor (GHR). Once activated, the GHR initiates JAK2/STAT5 signaling resulting in IGF-1 production. Knocking down GH signaling throughout the entire lifespan in model organisms is associated with an extension of healthspan and lifespan. This appears to be true for humans as well, as centenarians have higher frequencies IGF1R gene polymorphisms that result in altered IGF-1 signaling, and individuals with mutations in the growth hormone receptor gene that give rise to Laron dwarfism have decreased rates of age-associated pathologies, including diabetes and cancer [2]. However, reduced GH signaling in the post-juvenile period appears to have the opposite effect on healthspan and lifespan. A reduction in circulating IGF-1 in adulthood is a major risk factor for the development of many age-associated pathologies, including non-alcoholic fatty liver disease, diabetes, osteoporosis, sarcopenia, cardiovascular disease, dementia, and Alzheimer’s disease [3].

Levels of circulating GH and IGF-1 peak in early adulthood, then decline throughout the remaining lifespan in a process called somatopause. The mechanisms surrounding the natural reduction in GH and IGF-1 are not clearly understood. Recently, it was shown that the mir-465 family of miRNAs is upregulated with age in mouse liver and can directly influence GH signaling by targeting the GHR mRNA [4]. The mir-465 family consists of five genes located on the X-chromosome: one copy of mir-465a, and two copies each of mir-465b and mir-465c. Expression of these miRNAs led to a reduction in the GHR at both the mRNA and protein level, and a subsequent reduction in activation of the JAK2/STAT5 signaling pathway resulting in reduced IGF-1 and IGF-1 binding protein 3 (IGFBP3) expression. These findings suggest that the upregulation of the mir-465 family may contribute to the decline in IGF-1 with age.

The mechanisms behind this increase in miRNA expression has yet to be elucidated. The mir-465 family is part of a cluster of 18 miRNAs that are normally tightly repressed in liver. However, all 18 miRNAs in this cluster are upregulated with age [4]. This cluster is located in a highly heterochromatic region of the X-chromosome that is rich in repetitive elements and devoid of other coding sequences, consistent with constitutive heterochromatin. Chromatin structure undergoes a massive reorganization with age, with a closing of euchromatic regions and an opening of heterochromatic regions [5–8]. These changes in chromatin states correlate with changes in expression in these regions. Retrotransposons and other repetitive elements that are located in regions of constitutive heterochromatin increase in expression with age, likely due to the opening of the heterochromatin [6,7]. Relaxation of heterochromatin can lead to expression of miRNA genes located in these regions. Given that the X-chromosomal cluster is located a highly heterochromatic region, this mechanism may be responsible, at least in part, for the age-associated increase in expression of the 18 miRNAs, including mir-465 family. The miRNAs located in this cluster are predicted target hundreds of potential mRNAs, therefore, their expression may have broad reaching consequences on cellular function.

Currently, very little is known about the targets of this cluster of miRNAs. Only one other miRNA in the cluster, mir-470, has been studied thus far. It is expressed in the hippocampus of the long-lived Ames Dwarf mouse, where it leads to a reduction in the IGF-1 receptor. Bioinformatic analyses predict that three additional miRNAs in the X-chromosomal cluster (mir-743a, mir-871, and mir-881) may target IGF-1 [4], signifying that the age-associated up-regulation of this cluster of miRNAs may contribute to the dysregulation of the somatotropic axis. GH signaling is highly intertwined with the PI3K-Alt signaling network, which regulates cell metabolism, proliferation, survival and growth. Alterations in PI3K-Akt signaling has been implicated in the onset of many age-associated pathologies, including cancer, diabetes, and neurodegenerative conditions. Bioinformatic analyses predict 14 of the miRNAs in the X-chromosomal cluster have potential targets in 69 genes involved in PI3K-Akt signaling [4], suggesting that the 18 miRNAs may work together to contribute to the dysregulation of PI3K-Akt signaling with age, including GH signaling. Therefore, the age-associated upregulation of the X-chromosomal cluster of miRNAs may have impacts beyond GH signaling by influencing multiple pathways involved in cell growth and metabolism. Future studies investigating additional targets of these miRNAs will give insight into the molecular mechanisms involved in the development of aging phenotypes and pathologies, and may provide potential therapeutic targets to counteract these impacts.
